# Modeling the Energy Consumption of R600a Gas in a Refrigeration System with New Explainable Artificial Intelligence Methods Based on Hybrid Optimization

**DOI:** 10.3390/biomimetics8050397

**Published:** 2023-08-30

**Authors:** Sinem Akyol, Mehmet Das, Bilal Alatas

**Affiliations:** 1 Software Engineering Department, Engineering Faculty, Firat University, Elazig 23279, Turkey; balatas@firat.edu.tr; 2 Mechatronics Engineering Department, Engineering Faculty, Firat University, Elazig 23279, Turkey; m.das@firat.edu.tr

**Keywords:** vapor compression cooling system, energy consumption, R600a, explainable artificial intelligence, intelligent hybrid optimization

## Abstract

Refrigerant gases, an essential cooling system component, are used in different processes according to their thermophysical properties and energy consumption values. The low global warming potential and energy consumption values of refrigerant gases are primarily preferred in terms of use. Recently, studies on modeling properties such as compressor energy consumption, efficiency coefficient, exergy, and thermophysical properties of refrigerants in refrigeration systems with artificial intelligence methods has become increasingly common. In this study, a hybrid-optimization-based artificial intelligence classification method is applied for the first time to produce explainable, interpretable, and transparent models of compressor energy consumption in a vapor compression refrigeration system operating with R600a refrigerant gas. This methodological innovation obtains models that determine the energy consumption values of R600a gas according to the operating parameters. From these models, the operating conditions with the lowest energy consumption are automatically revealed. The innovative artificial intelligence method applied for the energy consumption value determines the system’s energy consumption according to the operating temperatures and pressures of the evaporator and condenser unit. When the obtained energy consumption model results were compared with the experimental results, it was seen that it had an accuracy of 84.4%. From this explainable artificial intelligence method, which is applied for the first time in the field of refrigerant gas, the most suitable operating conditions that can be achieved based on the minimum, medium, and maximum energy consumption ranges of different refrigerant gases can be determined.

## 1. Introduction

Vapor compression refrigeration systems are used in various fields, such as food preservation in domestic settings, large-scale industrial food storage applications, and air conditioning applications to provide thermal comfort in spaces. It is estimated that approximately 1.5 billion household refrigerators are in operation in the world today, and it is also estimated that 17% of the world’s electricity consumption is spent on the refrigeration sector, 45% of which is used in residential applications [1]. This situation highlights the importance of making some improvements to reduce energy consumption in cooling systems.

Various approaches have been proposed to improve the energy efficiency of cooling systems, such as improvements in heat exchangers, the use of alternative refrigerants, the commissioning of high-efficiency compressors, the use of improvements in insulation processes, and the implementation of modified refrigeration cycles [2].

Some studies have been conducted to determine performance parameters such as compressor power consumption and COP of the refrigeration system operating with new-generation hydrocarbon-type (HC) refrigerants. In these studies, new-generation HC refrigerants caused a reduction in compressor power consumption compared to old-generation refrigerants and provided higher COP value in the refrigeration system [3]. Several studies have been conducted on different refrigerants in vapor compression refrigeration systems to examine compressor energy consumption. The parameters considered in these studies are condenser–evaporator operating temperatures and compressor inlet–outlet pressures.

In his study, Rashed examined the COP values of five different refrigerants to express energy consumption at different condenser operating temperatures (38 and 45 °C) in a vapor compression refrigeration system (VCRS). He stated that the least energy consumption was obtained for R600a gas [4]. Madeira et al. examined the compressor power consumption of R134a and R600a gases at VCRS for the same condenser (32 °C) and evaporator (−9 °C) outlet temperatures. In all cases, R600a gas showed less power consumption characteristics [5]. Soni et al. compared the compressor power consumption values of five refrigerants at different evaporator temperatures (−9, −8, −7, −6, and −5 °C) in a VCRS. They showed that the lowest consumption value belonged to R600a gas at all five different evaporator temperature values [6]. Rasti et al. investigated the energy consumption of three different refrigerants at different condenser and evaporator operating temperatures in a domestic VCRS. They experimentally showed that the lowest energy consumption value was obtained for R600a gas [7]. Sánchez et al. investigated the compressor power consumption values of six refrigerants at six different evaporator and condenser temperature operating conditions in a VCRS. From their experimental research, they explained that the gas with the lowest compressor power consumption value was R600a [8]. Babarindea et al. examined the compressor energy consumption of R134a and R600a gases in VCRS. Different volume fractions of R600a gas and nanoparticulated R600a gas exhibited lower compressor power consumption [9]. Elakhdar et al. investigated the effects on compressor power consumption by examining the saturation pressures of R134a, R290, and R600a refrigerants at different operating temperatures in a VCRS. R600a, with the lowest pressure, consumed less energy [10]. In another study by Rasti et al. [7], the refrigerator test bench with an HFC-type compressor consumed 7% less power using R600a instead of R134A, while it consumed 18.7% less power with an HC-type compressor.

When studies on R600a refrigerant in the literature were examined, it was seen that this gas provides low compressor consumption. Studies have shown that the parameters affecting compressor power consumption are condenser and evaporator operating temperatures. For this reason, compressor power consumption values of R600a gas were examined in experiments conducted at different evaporator–condenser temperatures, and different pressures and datasets were obtained during the experiments. Transparent models expressing the compressor power consumption values of R600a were produced using these datasets and explainable artificial intelligence methods.

In artificial intelligence studies related to refrigeration systems, different artificial intelligence methods, especially ANN (artificial neural network) and ANFIS (adaptive neuro-fuzzy inference system), have been applied to model compressor power consumption and energetic and exergetic performance parameters of different refrigerants. Recently, the modeling of various parameters (coefficient of performance, compressor power consumption, refrigerant gas temperature, etc.) in refrigeration systems with artificial intelligence methods has become very popular [11]. Ghanbarpour et al. modeled the COP, cooling capacity, and carbon footprint of R449A and R404A refrigerants in a vapor compression refrigeration system with ANN. They stated that the error value between the model and experimental results did not exceed 10% [12]. Maiorino et al. used ambient temperature and system set temperature as input data to model the energy consumption of a VCRS system with ANN. They calculated that the MAPE value between experimental and ANN values was 3.8 [13]. Belman-Flores et al. estimated the energy consumption of a VCRS with the help of ANN using condenser and evaporator temperature values. They found the relative error between the predicted and experimental values to be 0.05 [14]. Asensio-Delgado et al. used machine learning to predict the solubility of high GWP refrigerants in ionic liquids in refrigeration systems. The average RMSE value of the model they obtained was 0.04 [15]. Yıldırım and Şahin estimated the energy and exergy performances of new-generation refrigerants (R515A, R516A, and R515B) in a VCRS with an adaptive-network-based fuzzy inference system. The average MAPE results of the predictive model for energy and exergy values of the three new-generation refrigerants were 5.7% and 5.5%, respectively [16].

Artificial intelligence methods for the abovementioned cooling systems are black-box approaches that, while better in accuracy values, can be insufficient in terms of explainability of the model. In black-box approaches, it is unknown how components (inputs) affect the goal value (output). Extensive literature studies on artificial intelligence show that practically all of the studies are black-box systems. Despite their excellent accuracy, these systems’ models are woefully inadequate in terms of explainability, interpretability, and comprehensibility. As a result, intelligent classification models with high precision, accuracy, recall, and interpretability are required. The motivation for this study, which marks a difference from other studies in the literature, is to propose a new model based on automatic transparent rules based on explainable optimization that expresses the compressor energy consumption values of R600a gas in refrigeration systems. The model is obtained using experimental data. It is important to note that this dataset has different operating temperatures, pressure values, mass flow rates, and compressor power consumption values of R600a. Therefore, using a common dataset for this refrigerant and generating models with this dataset increases the applicability of the generated models to other refrigeration systems operating with different refrigerants and makes the current study different from others in the literature.

**Figure 1:**
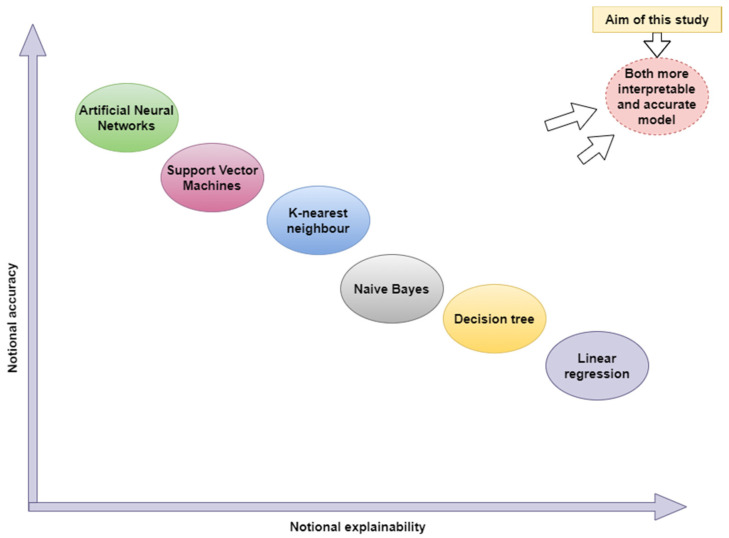
Motivation of the study.

Many classification models and architectures employed for the problem in focus are commonly referred to as “black boxes” due to their complex internal structure and inability to provide precise information to arrive at a decision. In general, the best models may have the least explainability, and the worst models may have the most explainability. This study is the first attempt at developing an intelligible, comprehensible, and interpretable transparent artificial intelligence method for modeling the energy behavior of R600 gas in a VCRS by balancing and optimizing both explainability and accuracy. Figure 1 depicts accuracy vs. explainability of the main artificial intelligence classification algorithms [17]. The models are ranked on the X-axis based on their interpretability, and the relative accuracy of these methods is mentioned on the Y-axis. Neural networks are at one end of the spectrum, achieving the highest accuracy but not being comprehensible and interpretable. The models where knowledge is most directly represented are located at the other side of the spectrum on the right. The current work aims to develop an optimization-based novel intelligent classification system with excellent explainability and accuracy capabilities.

The primary contributions of this work are listed as follows:

- The problem of obtaining models that determine the energy consumption values of R600a gas according to operating parameters is considered for the first time as an optimization problem with the goal of more successfully conducting classification in terms of various metrics.
- A hybrid intelligent optimization method that can be efficiently used for many search and optimization problems is proposed.
- A common dataset for this refrigerant is considered as search spaces, and a hybrid intelligent optimization method that combines constricted particle swarm optimization and tangent search algorithm is proposed by modifying the search strategy of a successful classification model to determine the compressor energy consumption values of R600a gas in refrigeration systems.
- The proposed hybrid intelligent optimization algorithm is adapted to function as an explainable, interpretable, comprehensible, and understandable classification rule mining method for the first time. By overcoming the difficulty of constructing a comprehensive optimal model while maximizing the performance metrics, the proposed hybrid intelligent optimization algorithm is implemented as a direct interpretable classification methodology for the first time.
- The proposed methodology avoids data preprocessing, such as fuzzyfication, discretization, and so on, while still identifying suitable intervals for features without changing or manipulating the data during the development of the classification model.
- The proposed intelligent optimization-based classification model is compared with legacy and state-of-the-art supervised classification methods. Although the proposed interpretable algorithm is new, it can achieve better results in many metrics by surpassing other methods.
- The proposed approach provides an important innovation in determining the optimum operating conditions to obtain the minimum energy consumption conditions of different refrigerants.

For R600a refrigerant gas, experiments were carried out at different condenser and evaporator operating temperatures in VCRS, and datasets consisting of measured parameters were obtained. Using this dataset, optimization-based, transparent, and explainable rules for the energy consumption of R600a gas in a refrigeration system under different operating conditions were obtained. The experimental setup, the measured parameters, and the artificial intelligence method are described in detail in the next sections.

## 2. Materials and Methods

### 2.1. Experimental Setup and Test Conditions Procedures

**Figure 2:**
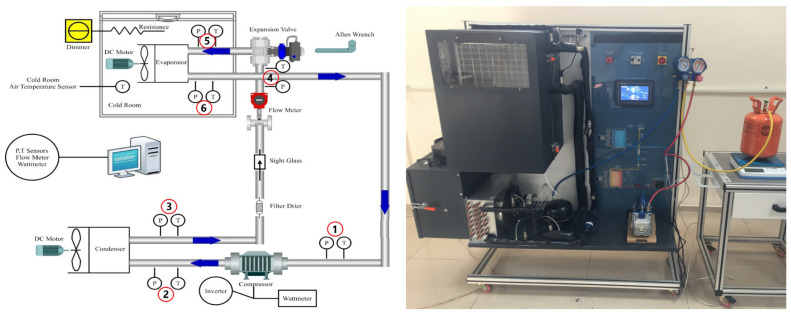
Schematic and real image of experimental vapor compression refrigeration system and measurement points.

A schematic of the experimental setup is depicted in Figure 2. A vapor compression refrigeration system mainly consists of a hermetic compressor originally designed for R600a and R290, an evaporator, a condenser, and an expansion valve. Polyolester oil was used in the compressor for both of the refrigerants. The compressor of the system works with an inverter so that the rotational speeds can be adjusted. The evaporator and condenser fans have inverters too. In this way, the evaporator and condenser temperatures can be set to desired values.

In this study, which is different than most studies in the literature, evaporator temperature adjustments were performed using not only the resistor connected to the dimmer but also the inverter of the evaporator fan. In addition, the changing air flow amount due to the inverter of the condenser fan made it possible to set the condenser temperatures according to the desired situation. The system also included a filter drier, a sight glass, a flow meter, and a vacuum pump for refrigerant replacement. Heat load in the cold room was supplied using an electrical resistor. The dimmer was used as the voltage regulator of the electrical current passing on the resistor. R600a refrigerant gas was used in the cooling system. Detailed properties of this gas are explained under the heading below.

#### R600a Gas Properties

The use of HC-based R600a refrigerant, which has no harmful effects on the environment, is becoming widespread in Europe, especially in domestic refrigerators. Resizing of the system design and components working with R600a refrigerant and investigation of the power consumption values are critical for cooling technology and a sustainable environment. The environmental impacts and thermodynamic properties (ASHRAE values) of R600a gas are tabulated in Table 1.

All sensors and measurement points are also seen in Figure 2. There were six pressure and temperature sensors at the inlet and exit of the main components, which were directly in contact with the refrigerant passing through copper pipes. There was a flow meter to obtain the mass flow rate of the refrigerants. The wattmeter available in the system enabled the compressor’s power consumption to be measured. All of the data were recorded in a computer using a data logger and PLC system. The symbols, units, and minimum and maximum ranges of the parameters measured during the experiments are given in Table 2. The technical characteristics of the measurement instruments are given in Table 3.

**Figure 3:**
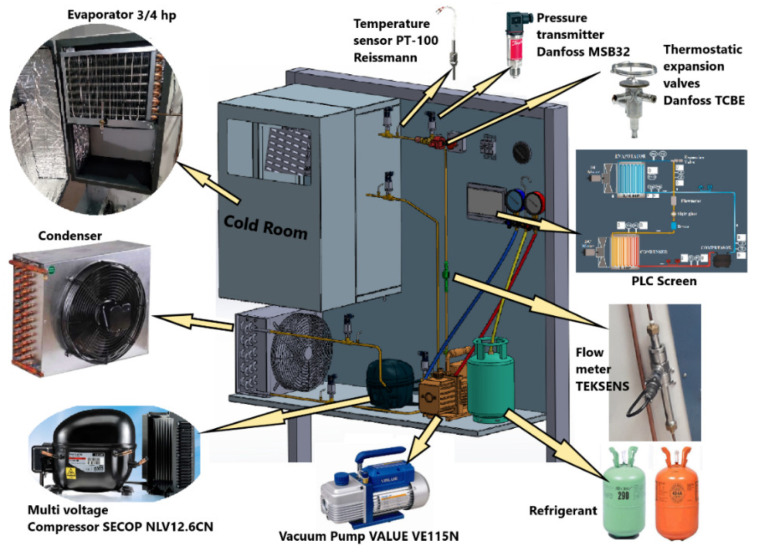
Experimental setup and system equipment.

The design of the experimental setup with all measurement devices and system equipment is shown in Figure 3. Brands and images of the utilized pressure and temperature sensors, expansion valve, flow meter, vacuum pump, and compressor can be seen from this figure. The experimental setup was designed for completely autonomous operation. All of the devices in the system were able to be controlled via the PLC screen, and measured data were able to be monitored instantly. Due to PLC automation, evaluating and processing of measured data have become easier and more practical.

Table 4 includes the operational parameters used in this study. Superheating degree is the difference between the saturation temperature corresponding to the measured pressure at the exit of the evaporator and the directly measured temperature by the sensor at the exit of the evaporator. The superheating degree was adjusted with a screw of the expansion valve using an Allen wrench. The subcooling degree is the difference between the saturation temperature corresponding to the measured pressure at the outlet of the condenser and the directly measured temperature by the sensor at the outlet of the condenser. In this study, the maximum variation of superheating degree was ±1.5 °C, and the subcooling degree changed with respect to operational conditions. In order to determine the evaporator and condenser operating temperature ranges during the experiments, all evaporator and condenser temperature data obtained during the period until the cooling system reached continuous regime conditions as a result of the expansion valve adjustments were averaged. Thus, four different evaporator and condenser operating temperatures were determined.

### 2.2. Dataset Creation Procedure

Intelligent hybrid-optimization-based classification methods were used to obtain explainable artificial intelligence models that express the compressor power consumption values of R600a gas in the refrigeration system. The datasets obtained from the parameters measured in Table 2 were used in these methods. All of the data transferred through data transfer in the PLC system in the experimental set were numerical data. Normalization or standardization applications were not applied to the dataset obtained as a result of the experiments. From the experiments, a dataset containing 7500 numerical values consisting of 15 attributes, including the parameters specified in Table 2, was created. Within this dataset, the compressor power consumption ranges were determined as follows: high consumption, ≥250 W; medium consumption, 200–250 W; low consumption, ≤200 W. While determining these ranges, the operating conditions in Table 4 were considered.

### 2.3. Artificial Intelligent Procedure

A novel hybrid metaheuristic algorithm that combines constricted particle swarm optimizer (CPSO) [18] and tangent search algorithm (TSA) [19] was developed and adapted for the focused problem.

#### 2.3.1. Hybrid Method

A population (swarm) in PSO is made up of particles, each of which represents a potential solution to the focused optimization problem. Each swarm particle has its own velocity and position and moves in a D-dimensional search space. The current velocity of each particle is defined by its previous velocity and the distance between the particle’s current position and the position where it has achieved its best fitness so far (called the personal best), denoted by *pbest*, and the position where the particle has achieved the best fitness (called the global best), denoted by *gbest*. The velocity of a particle is updated using the $v_{id}$ *pbest* and *gbest* values as shown in Equation (1).

(1) $$v_{id}=v_{id}+\varphi_{1}rand_{1}\left(pbest_{id}-p_{id}\right)+\varphi_{2}rand_{2}\left(nbest_{d}-p_{id}\right),\;\;\forall \;j=1,2,\ldots ,d$$

Here, $rand_{1}$ and $rand_{2}$ represent a random number between 0 and 1, and $p_{id}$ represents the current position of the *i*-th particle for the *d*-th dimension. The values $\varphi_{1}$ and $\varphi_{2}$ are constant numbers. After the particle’s velocity value is updated, its position value is also updated using Equation (2).

(2) $$p_{id}=p_{id}+v_{id},\;\forall \;j=1,2,\ldots ,d$$

Researchers have analyzed the performance of PSO using several variations and the constriction factor model, and the experimental findings showed that PSO utilizing the constriction factor and restricting the maximum velocity to the maximum location on each dimension performed better [20]. Therefore, Equation (3), which is its improved version, was used instead of Equation (1).

(3) $${\vec{V}}_{id}=\kappa \left(v_{i1}+\varphi_{1}rand_{1}\left(pbest_{id}-p_{id}\right)+\varphi_{2}rand_{2}\left(nbest_{d}-p_{id}\right)\right),\;\forall \;j=1,2,\ldots ,d$$

Here, the *κ* value is calculated using Equation (4). Typically, *φ*_1_ + *φ*_2_ is set to 4.1, and *K* is thus 0.729.

(4) $$K=\frac{2}{\left|2-\left(\varphi_{1}+\varphi_{2}\right)-\sqrt{{\left(\varphi_{1}+\varphi_{2}\right)}^{2}-4\left(\varphi_{1}+\varphi_{2}\right)}\right|}$$

**Figure 4:**
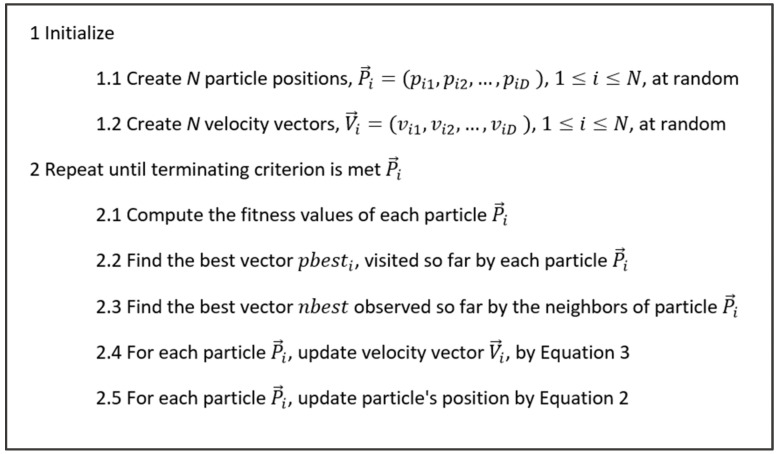
Pseudo-code of CPSO.

The main steps of constriction PSO (CPSO) is summarized in Figure 4.

In solution search and optimization strategies, high exploration and exploitation abilities are necessary. The purpose of the exploration phase is to thoroughly research the search space and discover the most promising prospective solutions. The exploitation phase is intended to direct the search process to the best possible option for the population. By correctly balancing exploration and exploitation performance, a metaheuristic method’s accuracy and speed of convergence can be improved. By combining an algorithm with strong exploitation but weak exploration with an algorithm with strong exploration but weak exploitation, a new hybrid algorithm that is stronger than these two algorithms can be created.

Because of the simplicity of the learning technique, the original PSO and CPSO have several flaws, including trapping in local optima, quick loss of diversity, and a poor balance of exploitation and exploration, particularly for many complex optimization problems [21]. In this study, to overcome these shortcomings of PSO and CPSO and to give more importance to exploitation capability in CPSO, TSA [19] is integrated and a new hybrid method that can be adapted for complex search and optimization problems is proposed. In order for the metaheuristic algorithm to perform well, the exploration and exploitation stages must work in harmony with each other. In this study, we aim to show better performance by applying TSA’s local minimum escape procedures to the PSO, whose exploitation stage is strong but whose exploration stage is weak.

TSA has recently been proposed as a new mathematics-based metaheuristic algorithm originating from the mathematical tangent function. This function offers a great capacity to explore the search space. The variation of this function between −∞ and +∞ and the periodicity of this function help to achieve a good balance between exploration and exploitation. In TSA, the entire update equation is governed by a global step of the form “(*θ*)step × tan(*θ*)”. Here, the tangent function plays the role of a flight function, as in the Levy flight function, which is why it is called tangent flight. TSA is made up of three primary components: an exploration search component that assures good exploration, an intensification search component that guarantees additional intensification search around the optimum solution, and an escape technique that is used to escape from local minima.

TSA includes a mechanism that uses a specific procedure to escape the local minimum stagnation problem. The procedure consists of two parts that are executed with a probability value. In each iteration, a random agent search is selected. This selected search agent is updated using either Equation (5) or (6) with equal probability.

(5) $$p_{id}=p_{id}+\tan\left(\theta \right)\times \left(upper\;bound-lower\;bound\right)$$

(6) $$p_{id}=p_{id}+R\cdot \;\left(nbest_{d}-p_{id}\right)$$

Equation (7) is used to calculate the *R* value.

(7) R=10×sign/log(1+(iteration number))

**Figure 5:**
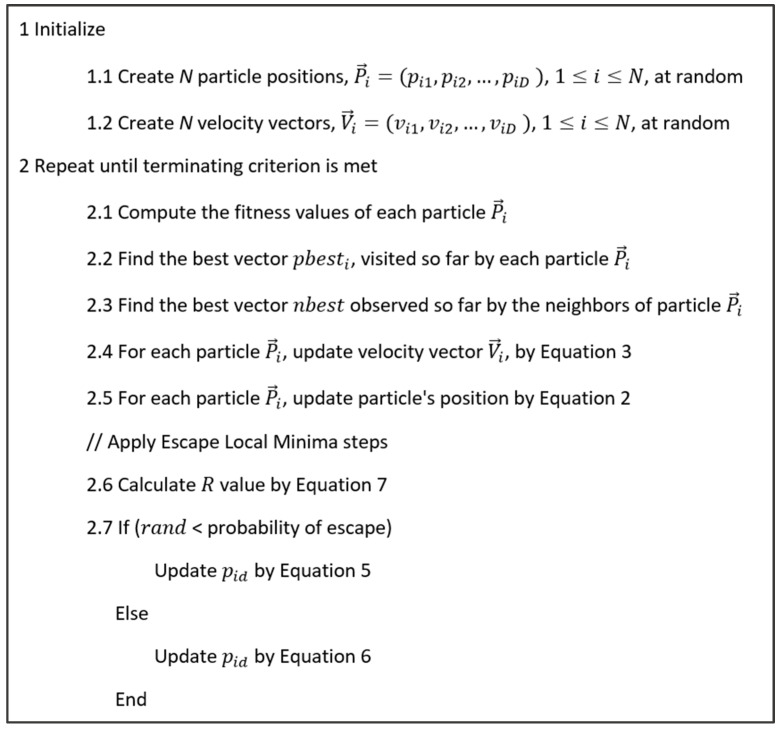
Pseudo-code of proposed hybrid CPSO-TSA model.

In the proposed hybrid constriction PSO tangent search algorithm (CPSO-TSA) approach, the escape local minima step of TSA is integrated into the end update procedure of CPSO according to a predetermined probability in order to achieve better results. The pseudo-code of the proposed hybrid CPSO-TSA model is shown in Figure 5.

#### 2.3.2. Adaptation of Hybrid Method to the Problem

##### Representation Type of CPSO-TSA for the Problem

**Figure 6:**
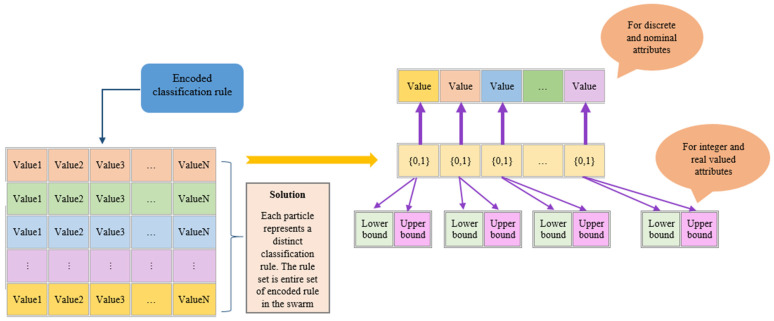
Encoding type of hybrid CPSO-TSA.

In this method, each particle is allowed to represent a unique classification rule for the explainable model. Interpretable and comprehensible classification rules are conditional clauses with two parts: antecedent and consequent. The former is a set of logical tests, while the latter specifies the class that applies to instances covered by this rule. The range [0.0, *t*] with 0.0 < *t* < 1.0 is used to standardize all attribute values. *t* is a user-predefined value that denotes the indifference threshold; a greater value causes the associated attribute test to be omitted. This normalized value is utilized in both the normalized real data and the space dimensions, preventing the need for expensive conversions to find attribute matching. The best shot at reducing the temporal complexity of the system involves using this normalized value throughout the real data, rule/dimensions, particle moving, and attribute matching processes [22]. In the proposed hybrid CPSO-TSA, each encoded rule consists of *n* parts, each of which contains 3 variables. One part is used for representing the inclusion or exclusion of the related attributes. The other two parts represent the lower bound and upper bound. This representation scheme resembles the encoding type used in [22]. The encoding type of CPSO-TSA is illustrated in Figure 6. All swarms are executed for the known class attribute. That is why, the class attribute is not encoded in the particles.

**Figure 7:**
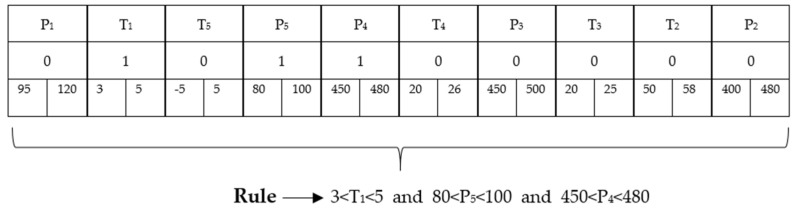
A representative rule and its expression.

Figure 7 shows a representative rule and its expression. P_1_–P_5_ and T_1_–T_5_ expressions represent the parameters in the cooling data. The values of 0 and 1 in the second row represent whether these parameters will be included in the rule. A value of 0 means that it is not included in the rule, and a value of 1 means that it is included in the rule. In the third row, it represents the lower limit and upper limit that each parameter can take in the rule. Accordingly, the obtained rule is expressed as follows.

##### Fitness Function

To establish points of reference for the training phase of the proposed hybrid CPSO-TSA, rules must be assessed during the training process. The rule assessment function must evaluate not just examples that have been correctly classified but also those that have yet to be classified and those that have been incorrectly classified. The fitness of particles is calculated according to Equation (8).

(8) $$itness\left(particle\right)=\{\begin{array}{cc} \frac{TP}{FN+TP}\times \frac{TN}{FP+TN}, & 0.0\leq value_{i}\leq 1.0,\;\forall i\in dimension\\ -1.0, & otherwise \end{array}$$

In this equation, *TP*, *FP*, *TN*, and *FN* represent true positives, false positives, true negatives, and false negatives, respectively. This equation penalizes a particle that has gone outside of its allowed values by assigning it a negative value (−1.0) and forcing it to return to the feasible search space for the explainable classification model.

##### Explainable Rule Set Construction and Evaluation

The rule creation approach for the explainable classification model is essentially a divide and conquer strategy. When given a training set, it conducts the interpretable rule mining process to find the best rule for the dominant class in the training data. When this rule is discovered, a pruning process in which extraneous attribute tests are deleted is applied. This is a straightforward procedure that deletes attribute tests iteratively if the quality of the produced rule is equal to or greater than that of the original rule. Instances that are correctly identified are subsequently deleted from the training set, and the comprehensible and interpretable rule mining phase for the explainable artificial intelligence model is run again. A sequential rule set is generated iteratively, and the covering method runs until only a predefined number of instances remain to categorize. This threshold criteria value is set as a percentage by the user and is generally set at 10%. A default rule in the form of “IF True THEN Class” is introduced to the constructed rule set to collect and classify occurrences that are not categorized by the prior rules. This rule has no attribute tests and predicts the same class as the one prevailing in the other occurrences.

To assess the accuracy of the comprehensible rules constructed from the proposed explainable model obtained by the rule set construction method, the k-fold method is used. Furthermore, splitting the data as train and test is also a general method for validation.

Each instance is given a state attribute. It is fairly simple and effective to partition the dataset into test and training sets and to (pseudo-) eliminate occurrences using this state value. “Train”, “Test”, and “Removed” values are assigned to the attributes. When a rule is returned from the classification rule mining procedure, it is pruned to delete extraneous attribute tests for comprehensibility. This is accomplished by deleting each attribute test iteratively whenever the newly discovered rule has the same or greater quality value as the original constructed rule.

**Figure 8:**
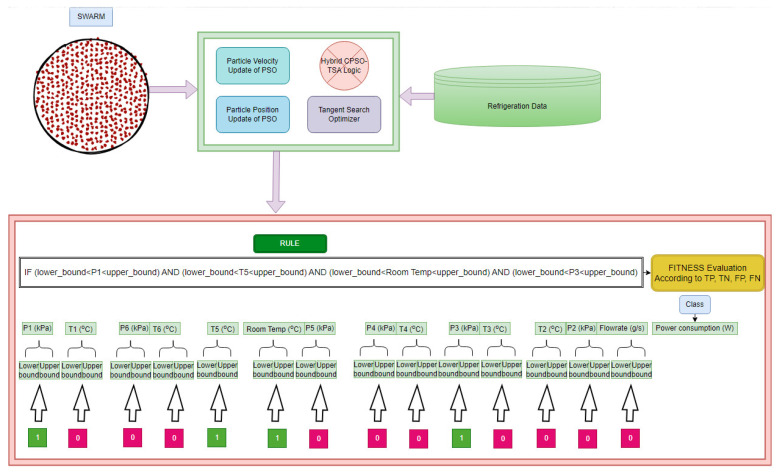
Illustration of the proposed hybrid CPSO-TSA and candidate representation form for the refrigeration data.

**Figure 9:**
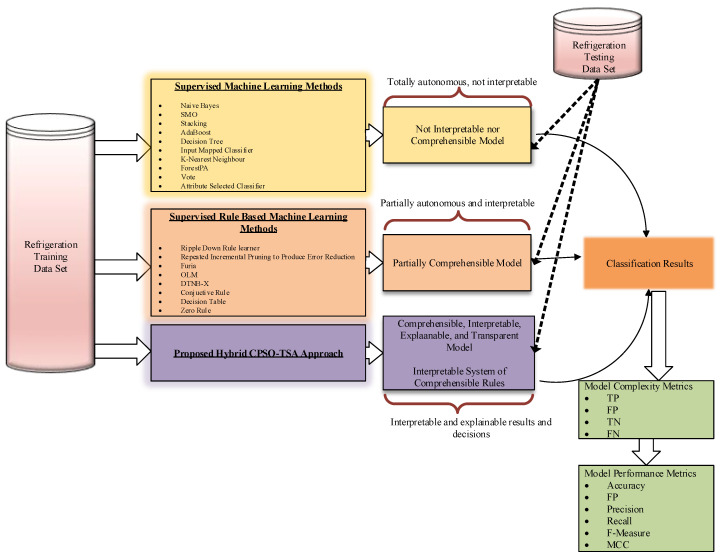
General structure of the system and methodology.

All processes and the candidate particle representation scheme employed in the proposed hybrid CPSO-TSA are illustrated in Figure 8 for a better understanding of the explainable model. Figure 9 depicts the major details of the approach and the system employed in the study.

## 3. Results and Discussions

In the present study, the compressor power consumption values of R600a gas in a VCRS under different operating conditions were investigated and modeled with explainable artificial intelligence methods. The experimental and model results obtained in the study are given below.

### 3.1. Experimental Results

**Figure 10:**
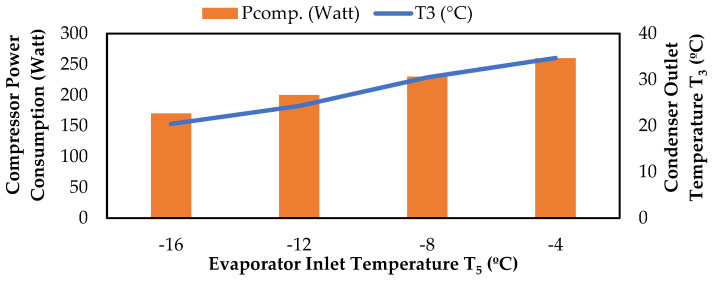
Change in compressor power consumption values according to T_3_ and T_5_.

**Figure 11:**
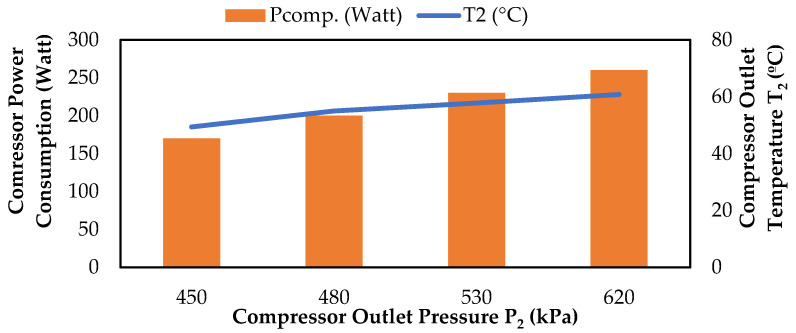
Change in compressor power consumption values according to P_2_ and T_2_.

**Figure 12:**
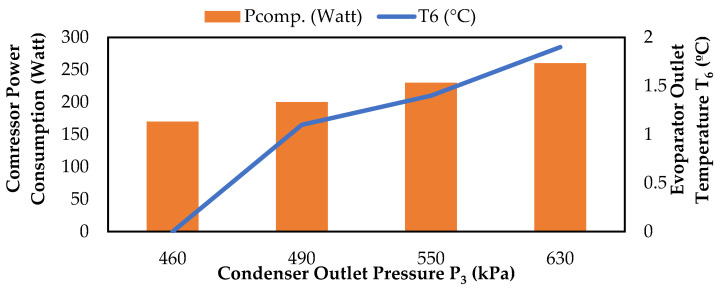
Change in compressor power consumption values according to P_3_ and T_6_.

The compressor power consumption values measured under the test conditions specified in Table 4 for R600a gas and the variation of these values according to the pressure and temperature values of the cooling system elements are given in Figure 10, Figure 11 and Figure 12. As shown in Figure 10, the compressor power consumption value increased as the evaporator temperature increased. This is because the compressor has to work harder to maintain the same level of refrigeration effect when the evaporator temperature is higher, which requires more energy input [23]. Likewise, the compressor power consumption value increased as the condenser temperature increased. This is because the compressor has to work harder to pump the refrigerant against the increased back pressure in the system. The compressor has to increase the pressure difference between the high-pressure side and the low-pressure side to maintain the same cooling effect, which increases the power consumption [24].

As shown in Figure 11, as the compressor outlet temperature and pressure values increased, the power consumption values of the compressor also increased. This is because the compressor has to work harder to compress the refrigerant to a higher pressure and temperature, which requires more energy. Additionally, if the compressor is working harder, it may also need to consume more power to overcome any frictional losses within the compressor [25].

As shown in Figure 12, the compressor power consumption value increased as the condenser outlet pressure increased. This is because the compressor has to work harder to overcome the increased pressure in the condenser and maintain the same level of refrigerant flow. This increased workload leads to higher energy consumption by the compressor [26].

### 3.2. Model Results

In this study, in order to compare the explainable results obtained from the CPSO and CPSO-TSA algorithms, the conjunctive rule, decision table, DTNB-X, FURIA, JRip, multiobjective evolutionary, OLM, OneR, PART, Ridor, and ZeroR algorithms with partially explainable results were used. In addition, the results were compared with the results obtained from the machine learning algorithms, which gave black-box results. The number of true positives (*TP*), number of false positives, number of rules, accuracy, precision, recall, F-measure, and MCC values were used to evaluate the results obtained from the algorithms. In the first stage, the results were obtained using the whole dataset as training data. Afterwards, the dataset was separated as training and test data using *k*-fold validation techniques. In this study, the *k* value was taken as 3 and 5. Population size was taken as 50 in the CPSO and CPSO-TSA algorithms. The termination condition of the algorithms was that the global best solution does not change for 30 iterations or the Euclidean distance between the global best solution and the other particles is less than 0.01.

Table 5 shows the explanatory model obtained for the cooling data from the CPSO-TSA algorithm proposed in this study. While the “Rules” column expresses the rules of the model, the “Class” column indicates the class to which this rule belongs. “TP” represents the number of true positives, and “FP” represents the number of false positives. According to this model, four rules were found, including two “medium”, one “low”, and one “high” class. It was predicted that those remaining outside the examples covered by these four rules belonged to the “low” class. The results of this model are shown in Table 5.

In VCRS, compressor power consumption studies have generally been carried out in the evaporator and condenser temperature pressure ranges. The explainable rules obtained in Table 5 show that the parameters on which the compressor power consumption depends for R600a gas are the same as the literature. According to the rules, the effects of condenser temperature (T_3_), evaporator temperature (T_5_), and compressor outlet pressure (P_2_) parameters on power consumption were also examined. However, although the condenser outlet pressure (P_3_) and evaporator outlet pressure (P_5_) also have an effect on power consumption, there is no study expressing the effects of these parameters. This shows that the obtained transparent rules contain interesting results.

Table 6 shows the results obtained from CPSO, CPSO-TSA, and partial explainable models when the whole dataset was considered as training data. From the table, it can be seen that the CPSO-TSA proposed in this study gave the highest number of true classified samples, accuracy, and MCC values. Similarly, compared to other algorithms, CPSO-TSA seemed to give a more reasonable number of rules. The lowest FP (false positive rate) and number of false classified samples values were also obtained from CPSO-TSA.

The results obtained from the proposed model and well-known machine learning algorithms when the whole dataset was considered as training data are shown in Table 7. As shown in the table, the highest accuracy, precision, and MCC values were obtained from the CPSO-TSA proposed in this study. Similarly, it can be seen that the lowest FP rate was also obtained from CPSO-TSA.

In order to demonstrate the effectiveness of the proposed model, the energy behavior of R600 gas in the test dataset was classified using the cross-validation method. For cross-validation, the *k* value was taken as 3 and 5. Table 8 shows the results obtained from the proposed method and partially explainable machine learning methods when *k* = 3 folds. As shown in this table, the highest accuracy value was obtained from the CPSO-TSA algorithm proposed with the part algorithm after the OneR algorithm. Similarly, when looking at the MCC value, CPSO-TSA gave the highest value after the OneR algorithm.

When *k* = 5 folds, the results obtained from CPSO, the proposed CPSO-TSA method, and partially explainable machine learning methods are shown in Table 9. According to the table, the CPSO-TSA proposed in this study gave the highest value after FURIA and OneR methods for the accuracy metric. Similarly, for the other metrics in the table, it can be seen that the CPSO-TSA, which produces an explainable model, gave very good results.

The error rates obtained when all the data were used as training data are given in Table 10. As can be seen from this table, the RMSE (root mean square error) value was found to be 0.3949 in the CPSO-TSA method proposed in this study. Promising results were obtained compared to other methods. When the MAE (mean absolute error) value was examined, it gave better results than methods such as conjunctive rule, decision table, PART, and ZeroR. When the kappa value was examined, among the 11 methods, it gave better results than the other methods except the PART algorithm.

Some studies in the literature that have modeled the energy consumption values examined in this study with artificial intelligence methods are shown in Table 11. In this study, the energy consumption of R600a gas in the system was modeled with the optimization-based explainable artificial intelligence method as well as the black-box methods conjunctive rule learner, decision table, DTNB, FURIA, OLM, OneR, PART, Ridor, and ZeroR. Some studies in the literature that have modeled the compressor power consumption values examined in this study with artificial intelligence methods are shown in Table 11. It can be seen that the model error results using black-box artificial intelligence methods are similar to the black-box model error results in the current study. Despite the excellent accuracy of the models in Table 11, the models of these systems are unfortunately insufficient in terms of explainability, interpretability, and comprehensibility.

**Figure 13:**
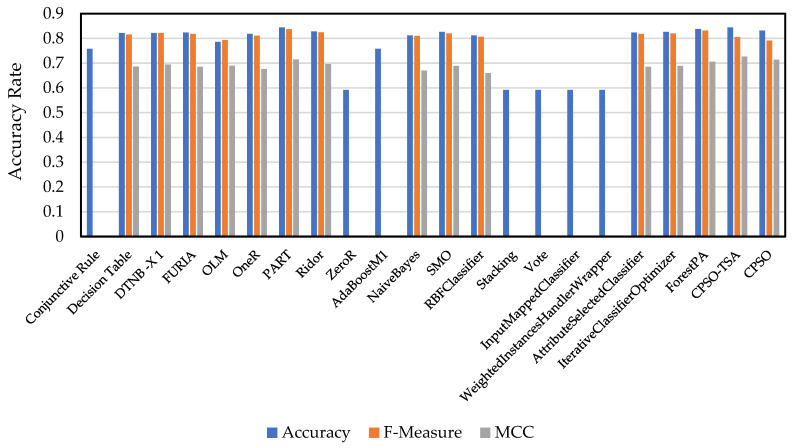
Accuracy, F-measure, and MCC bar graph of algorithms in Table 7 and Table 8 for 100% training data.

The bar graph of the accuracy, F-measure, and MCC values of the algorithms in Table 8 and Table 9 are shown in Figure 13 for 100% training data. As shown in the table, the CPSO-TSA method proposed in this study gave very successful results for these three metrics compared to other algorithms. It was observed that CPSO-TSA achieved 84.4% success in the accuracy metric and 80.54% in the MCC metric.

## 4. Conclusions

Refrigerant gases, which are used in different processes according to their energy consumption values and are a basic cooling system component, are preferred primarily in terms of their energy consumption values and low global warming potential. For a sustainable environment and refrigeration technology, it is of critical importance to resize and examine the power consumption values of the components working with the increasingly popular R600a refrigerant gas. In this study, for the first time, the classification of energy consumption values of R600a gas according to operating parameters was considered as an optimization problem. In addition, in order to increase the performance of the CPSO algorithm, a new intelligent hybrid optimization method called CPSO-TSA, in which TSA’s escape local minima steps are applied, was proposed. Refrigerant dataset was considered as search spaces, and a hybrid intelligent optimization method was proposed by modifying the automatic direct search strategy of a successful classification model to determine the compressor energy consumption values of R600a gas in refrigeration systems. This study modeled determination of the optimum operating conditions to achieve minimum energy consumption conditions for different refrigerants as an automatic rule-based optimization problem, which is a novel concept, and interesting and promising results were obtained from this new methodology using the designed proper fitness function and suitable representation scheme. All performance criteria for the proposed optimization based direct classification model will be simultaneously improved in the future.

This paper presents explainable and interpretable rules for the energy consumption of R600 gas using system parameters measured in a VCRS. There are valuable studies in the literature that model system irreversibility using ANN [33,34,35]. This study used direct compressor power consumption values as the basis for energy consumption values. In the future, performance parameters, including system irreversibility and power consumption, will be modeled with explainable artificial intelligence methods using different refrigerants. When the results were examined in general, the proposed hybrid CPSO-TSA model showed 84.4% success when used as training data, 80.6% when taken as *k* = 3 fold, and 79.67% when taken as *k* = 5. Compared to other algorithms, it gave very successful results. In addition, considering the error rates, it achieved good success by giving a very low value of 0.16 MAE. Similarly, it performed well, achieving 0.39 RMSE and 0.69 kappa statistics.

The proposed comprehensible, transparent, interpretable, and explainable artificial intelligence model provides an important innovation in determining the optimum operating conditions to obtain the minimum energy consumption conditions of different refrigerants. The proposed intelligent optimization-based interpretable methodology also seems to be successfully and easily adapted for numerous classification tasks. Different hybridization approaches, adaptive methods, and parallel versions of this method are other possible topics for further research.

## Figures and Tables

**Table 1:** Main properties of R600a.

Property	R600a
ODP	0
GWP	<1
Normal boiling point (°C)	−11.76
Critical temperature (°C)	134.7
Critical pressure (MPa)	3.64
Liquid density (kg dm^−3^) (0 °C)	4.257
Vapor density (kg m^−3^) (0 °C)	0.581
Liquid specific heat (kJ kg^−1^ K^−1^) (0 °C)	2.31
Vapor specific heat (kJ kg^−1^ K^−1^) (0 °C)	1.64
Liquid thermal conductivity (W m^−1^ K^−1^) (0 °C)	0.107
Vapor thermal conductivity (W m^−1^ K^−1^) (0 °C)	0.014
Liquid viscosity (μPa s) (0 °C)	199.33
Vapor viscosity (μPa s) (0 °C)	6.97
Oil type	Polyester

**Table 2:** Measured parameters and their features.

Measured Parameter	Symbol	Unit	Range
Compressor inlet pressure	P1	kPa	100~140
Compressor inlet temperature	T1	°C	−0.5~17.5
Condenser inlet/compressor outlet pressure	P2	kPa	450~800
Condenser inlet/compressor outlet temperature	T2	°C	37~65.7
Condenser outlet pressure	P3	kPa	460~810
Condenser outlet temperature	T3	°C	23.3~42.5
Expansion valve inlet pressure	P4	kPa	450~810
Expansion valve inlet temperature	T4	°C	23~43.5
Evaporator inlet/expansion valve outlet pressure	P5	kPa	100~190
Evaporator inlet/expansion valve outlet temperature	T5	°C	−16.4~−3.7
Evaporator outlet pressure	P6	kPa	100~160
Evaporator outlet temperature	T6	°C	−9.4~13.6
Cold room temperature	Tc	°C	−8.3~4.3
Mass flow rate (g/s)	m	g/s	0.69~2.41
Compressor power consumption	Pc	Watt	160~340

**Table 3:** Details of the measurement instruments.

Instrument	Brand	Accuracy
Pressure sensors	Danfoss	±0.3%
Temperature sensors	Reissmann	±0.5 °C
Flow meter	Teksens	±1%
Wattmeter	Entes	±1%

**Table 4:** Test conditions.

Operational Conditions	R600a
Evaporator temperatures (°C)	−16, −12, −8, and −4
Condenser temperatures (°C)	20, 25, 30, and 35
Average measured superheating degree	10.5
Average measured subcooling degree	8
Compressor speed (rpm)	1000

**Table 5:** Obtained explainable model consisting of interpretable rule set.

Rules	Class	TP	FP
If T_5_(°C) in [−10.099, −3.7] and P_3_(kPa) in [460.0, 551.469] and T_3_(°C) in [23.407, 42.5]	Medium	219	30
If T_6_(°C) in [−8.137, 13.9] and T_5_(°C) in [−16.4, −6.734] and P_3_(kPa) in [460.0, 716.739] and T_2_(°C) in [40.39, 56.542]	Low	113	37
If T_6_(°C) in [−3.118, 13.694] and P_5_(kPa) in [105.067, 190.0] and P_2_(kPa) in [456.931, 800.0]	High	60	5
If T_6_(°C) in [−8.411, 13.9] and T_2_(°C) in [37.0, 61.514] and P_2_(kPa) in [450.0, 572.679]	Medium	39	1
Exceptions to these rules	Low		

**Table 6:** Results obtained from explainable and partial explainable models in all training data.

Algorithm	Number of True Classified Samples	Number of False Classified Samples	Number of Rules	Accuracy	FP	Precision	Recall	F-Measure	MCC
Conjunctive Rule	379	121	2	0.758	0.237	?	0.758	?	?
Decision Table	411	89	13	0.822	0.138	0.850	0.822	0.816	0.686
DTNB-X 1	411	89	118	0.822	0.104	0.839	0.822	0.822	0.694
FURIA	412	88	5	0.824	0.141	0.849	0.824	0.818	0.685
OLM	393	107	302	0.786	0.080	0.880	0.786	0.793	0.690
OneR	409	91	1	0.818	0.143	0.846	0.818	0.811	0.676
PART	422	78	11	0.844	0.136	0.862	0.844	0.837	0.715
Ridor	414	86	11	0.828	0.122	0.846	0.828	0.825	0.696
ZeroR	296	204	-	0.592	0.592	?	0.592	?	?
CPSO-TSA	422	78	5	0.844	0.0964	0.8703	0.7898	0.8054	0.7264
CPSO	416	84	5	0.832	0.1006	0.8695	0.7808	0.7918	0.7137

**Table 7:** Results obtained from proposed model and machine learning algorithms.

Algorithm	Accuracy	FP	Precision	Recall	F-Measure	MCC
AdaBoostM1	0.758	0.237	?	0.758	?	?
NaiveBayes	0.812	0.124	0.824	0.812	0.81	0.67
SMO	0.826	0.138	0.851	0.826	0.82	0.689
RBFClassifier	0.812	0.156	0.833	0.812	0.807	0.66
Stacking	0.592	0.592	?	0.592	?	?
Vote	0.592	0.592	?	0.592	?	?
InputMappedClassifier	0.592	0.592	?	0.592	?	?
WeightedInstancesHandlerWrapper	0.592	0.592	?	0.592	?	?
AttributeSelectedClassifier	0.824	0.141	0.849	0.824	0.818	0.685
IterativeClassifierOptimizer	0.826	0.138	0.851	0.826	0.82	0.689
ForestPA	0.838	0.136	0.855	0.838	0.832	0.706
CPSO-TSA	0.844	0.0964	0.8703	0.7898	0.8054	0.7264
CPSO	0.832	0.1006	0.8695	0.7808	0.7918	0.7137

**Table 8:** The results obtained when *k* = 3 folds.

Algorithm	Accuracy	FP	Precision	Recall	F-Measure	MCC
Conjunctive rule	0.758	0.237	?	0.758	?	?
Decision table	0.788	0.171	0.810	0.788	0.782	0.619
DTNB-X 1	0.782	0.147	0.792	0.782	0.779	0.616
FURIA	0.794	0.169	0.803	0.794	0.785	0.623
OLM	0.338	0.278	0.532	0.338	0.255	0.092
OneR	0.818	0.143	0.846	0.818	0.811	0.676
PART	0.808	0.177	0.827	0.808	0.803	0.644
Ridor	0.800	0.150	0.810	0.800	0.795	0.641
ZeroR	0.592	0.592	?	0.592	?	?
CPSO-TSA	0.806033	0.193967	0.804433	0.7451	0.757033	0.649433
CPSO	0.7811	0.2189	0.797333	0.666467	0.651533	0.5585

**Table 9:** The results obtained when *k* = 5 folds.

Algorithm	Accuracy	FP	Precision	Recall	F-Measure	MCC
Conjunctive rule	0.758	0.237	?	0.758	?	?
Decision table	0.790	0.159	0.806	0.790	0.783	0.624
DTNB-X 1	0.796	0.139	0.805	0.796	0.793	0.639
FURIA	0.812	0.140	0.825	0.812	0.807	0.663
OLM	0.340	0.275	0.549	0.340	0.258	0.100
OneR	0.810	0.145	0.829	0.810	0.803	0.661
PART	0.790	0.175	0.793	0.790	0.786	0.612
Ridor	0.788	0.149	0.795	0.788	0.784	0.623
ZeroR	0.592	0.592	?	0.592	?	?
CPSO-TSA	0.79662	0.20338	0.78424	0.73568	0.72494	0.62782
CPSO	0.758	0.242	0.68758	0.66084	0.63576	0.53956

**Table 10:** Error values of results obtained from explainable and partially explainable models in all training data.

Algorithm	RMSE	MAE	Kappa Statistic
Conjunctive rule	0.3489	0.2446	0.5425
Decision table	0.2924	0.1856	0.6822
DTNB	0.3109	0.1495	0.6968
FURIA	0.3431	0.124	0.6852
OLM	0.3777	0.1427	0.6579
OneR	0.3483	0.1213	0.675
PART	0.2867	0.1644	0.7171
Ridor	0.3386	0.1147	0.6982
ZeroR	0.4309	0.3718	0
CPSO-TSA	0.3949	0.1556	0.6490
CPSO	0.4099	0.168	0.6998

**Table 11:** Summary of previous studies related to the subject of the study.

Modeled Parameter	Refrigerant Type	Methodology	Model Accuracy	Reference
COP and compressor power consumption	R134a	ANN	11.68% and 1.68% maximum deviations	[27]
Cooling capacity, COP, and	R404A	ANN	90% and 95% accuracy	[12]
compressor power consumption	R600a	ANFIS	R = 0.993	[1]
COP and compressor power consumption	R600a	ANN	11% and 4.2% MAPE	[28]
Compressor power consumption	R600a	MLP, SVM, and DT	6%, 4%, and 9% MAPE	[29]
COP and compressor power consumption	R600a/MWCNT nanolubricant	ANFIS	8.9% and 7.2% MAPE	[30]
Compressor power consumption	CO_2_/NH_3_	GA-LSSVM	0.08 RMSE	[31]
Compressor power consumption	R290/R600a mixture	Multiple regression and ANN	R^2^ = 0.96 and R^2^ = 0.993	[32]

## Data Availability

Data available on request due to restrictions, e.g., privacy or ethical. The data presented in this study are available on request from the corresponding author. The data are not publicly available. The data used in this manuscript belong to the experimental device in the Tokat project, number GOP 2020/122. Data will only be shared with special permission.
